# Real-world evidence: Telemedicine for complicated cases of urinary tract infection

**DOI:** 10.1371/journal.pone.0280386

**Published:** 2023-02-02

**Authors:** Natalie M. Daumeyer, Daniel Kreitzberg, Kathleen M. Gavin, Timothy A. Bauer

**Affiliations:** 1 Everly Health, Inc., Austin, Texas, United States of America; 2 University of Colorado Anschutz Medical Campus, Aurora, Colorado, United States of America; University of Catania: Universita degli Studi di Catania, ITALY

## Abstract

Telemedicine programs for the treatment of urinary tract infections (UTIs) offer an opportunity to reduce burdens on patients and providers. However, these programs are typically restricted to patients with uncomplicated UTIs. This real-world analysis evaluated treatment and resolution rates in a large-scale, national UTI telemedicine program inclusive of patients with uncomplicated and complicated UTIs. We conducted a retrospective analysis of data obtained from a commercially available telemedicine program for the treatment of UTIs among adult women in the US between 2017 and 2021 (n = 51,474). The primary outcomes were the number of women who presented with symptoms of uncomplicated UTI, complicated UTI, and vaginal infection; prescription use and antibiotic type; symptom resolution within seven days after appointment; and treatment failure or relapse. Most patients reported frequent urination (94.4%), urgency (94.5%), and dysuria (97.6%). Those with uncomplicated UTI symptoms represented the majority of patients (61.6%); however, a substantial number of patients (36.5%) also reported at least one symptom associated with a complicated UTI. One-fifth of patients (19.2%) reported at least one co-occurring symptom of vaginal infection or sexually transmitted infection. Across all treated patients, 94.0% received recommended antibiotics according to the clinical protocol. Of the treated patients who provided follow-up data (n = 3,521), 89.7% reported seven-day symptom resolution. Symptom resolution rates were similar between patients with uncomplicated UTI symptoms (90.8%) and complicated UTI symptoms (87.9%), and symptom resolution among all treated patients (89.7%) was similar to reports for in-person standard of care. These findings suggest that large-scale telemedicine programs for the treatment of UTIs can be effective in the treatment of complicated UTIs.

## Introduction

Urinary tract infections (UTIs) are some of the most common infections, accounting for millions of visits to healthcare facilities every year [1]. The use of urinalysis in emergency departments to diagnose suspected UTIs has increased from 23% in 2007 to 27% in 2016 [2]. During these visits, patients can spend more than two hours waiting for and receiving care, creating a burden for patients [3]. Using telemedicine as an alternative model of care for patients with UTIs would reduce the amount of time patients spend seeking treatment, while freeing up important resources in often over-burdened urgent care and emergency departments, as well as primary care clinics [4, 5]. UTIs make up 0.7% of outpatient services, according to projections from US registries [1]. Between 1996 and 2001, an average of 7 million women visited healthcare centers in the United States (US) for uncomplicated UTIs [6]. In 2007, there were 10.5 million ambulatory visits for UTIs in the US, accounting for approximately 1% of all ambulatory visits. Of those, 21.3% were to emergency departments [7]. Consultations for UTIs represent between 1% and 6% of all medical visits (~7 million visits and ~$1.6 billion annually) [8]. Previous research has shown that telemedicine treatment programs are effective for women presenting with uncomplicated UTI symptoms [9–15]. These programs, which largely rely on the application of inclusion/exclusion criteria according to self-reported symptoms and empirical use of antibiotics [16], are similar to in-person care and reduce time to recovery compared with diagnosis using urinalysis [16, 17]. However, a limitation of these programs is the exclusion of patients who self-report symptoms of complicated UTIs (e.g., fever, nausea) or vaginal infections (e.g., vaginal discharge, vaginal irritation) [9, 11–14]. Thus, it is unclear how effective or safe telemedicine programs can be for patients with these clinical profiles.

Women with symptoms of complicated UTIs (e.g., pyelonephritis) are usually referred to in-person care to receive intravenous antibiotics and/or undergo urinalysis to determine the appropriate antibiotic regimen [18, 19]. However, some complicated UTI cases can be managed at home without admittance to an emergency department. Researchers have thus begun to question whether higher-risk patients, such as those who report symptoms of pyelonephritis or more complicated UTIs, can be treated via telemedicine [15, 18, 20].

In a similar manner, women with vaginal symptoms are often referred to in-person care to rule out other diagnoses, such as sexually transmitted infections (STIs) and vaginal infections [16, 21, 22]. However, even cases of suspected STI may be managed by remote sample collection and telemedicine services [23]. Recent evidence has shown that vaginal discharge does not reduce the likelihood of a UTI [24], contradicting earlier studies advocating for in-person treatment for women with vaginal symptoms [16]. Thus, with proper symptom screening, which can be implemented virtually, a process for ruling in or out vaginal infections and STIs may be accomplished [25]. Although patients with symptoms of complicated UTI or vaginal infection may benefit from a telemedicine approach to care, current studies evaluating the value of telemedicine have excluded these patients. It remains unknown whether telemedicine provides an alternative, effective pathway to care without compromising on quality.

This study examined real-world data from a large telemedicine program that sought to provide treatment for women with UTI symptoms. This UTI telemedicine program is different from previously evaluated programs [15] in the following ways: 1) symptoms reported during the initial screening are included, 2) patients who reported symptoms of complicated UTI and vaginal infection were included, and 3) physicians were given discretion as to whether and how to treat women who reported symptoms of complicated UTI or vaginal infection. The primary outcome measures were the number of women who presented with symptoms of uncomplicated UTI, complicated UTI, and vaginal infection; prescription use and antibiotic type; symptom resolution within 7 days after appointment; and treatment failure or relapse.

## Materials and methods

### Ethics

This study is a retrospective analysis of real-world data from a nationwide commercial telemedicine program for the treatment of UTIs. This study met the requirements for a waiver of informed consent by the WCG Institutional Review Board (IRB00000533) because it does not meet the definition of human subjects research as defined in federal regulation 45 CFR 46.102.

### Study population and data collection

**Fig 1:**
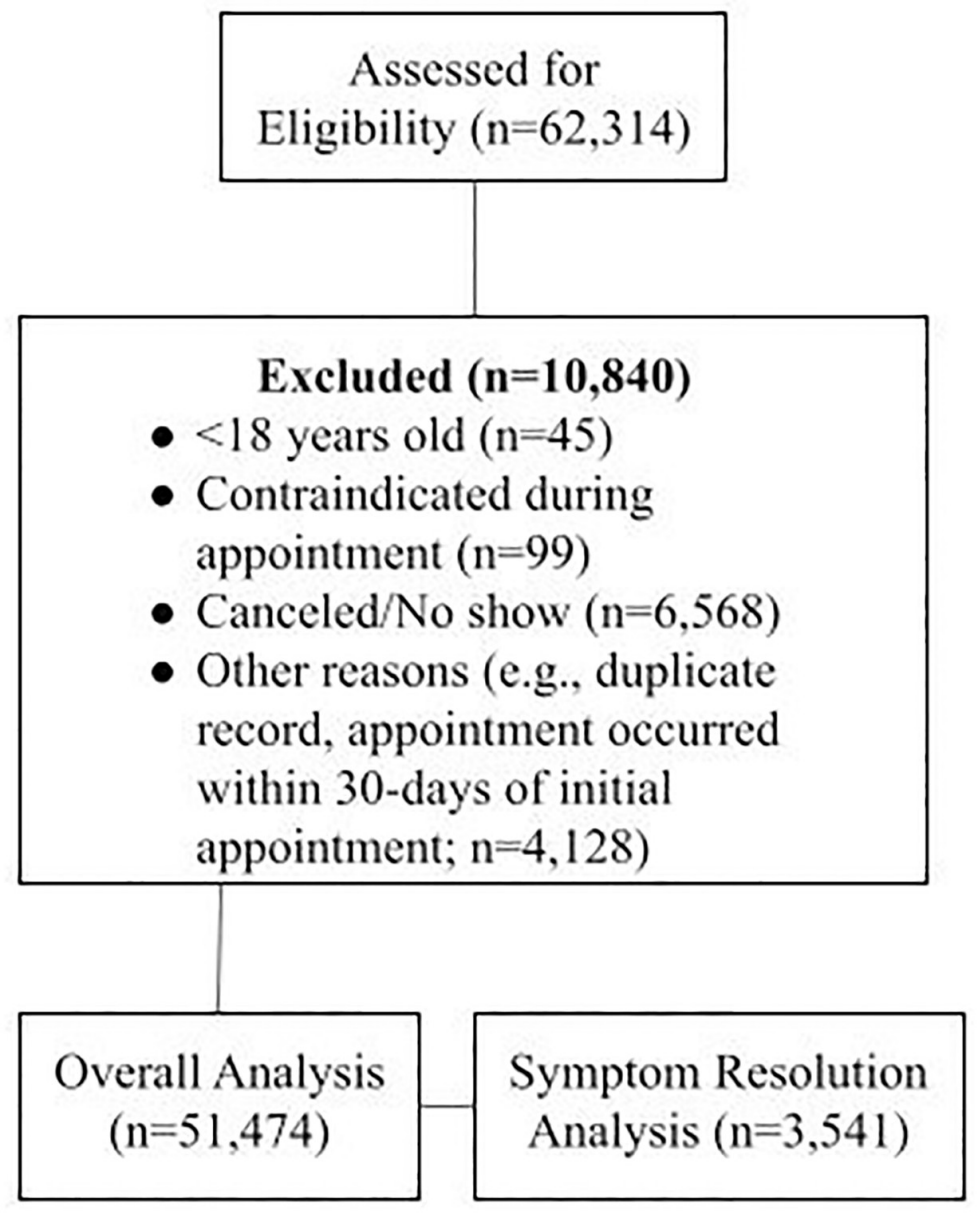
Study flow chart.

Individuals in the US seeking care for UTI symptoms between November 2017 and November 2021 completed a digital screening process to determine their eligibility for telemedicine services. Patients were excluded for the following reasons: younger than 18 years of age, male, pregnant or breastfeeding, immunocompromising conditions (e.g., HIV, lupus), history of a kidney condition, history of a urinary condition, history of urologic procedure, history of resistant organisms, or inpatient stay at a healthcare facility within the past three months. For this protocol, prior to the COVID-19 pandemic, women aged 65 years or older were excluded if they were currently taking more than five prescription medications. This exclusion was removed during the pandemic because of restricted access to in-person care. Cancelled/missed appointments, duplicate records, and follow-up appointments occurring within 30 days after the initial appointment were excluded from the analysis. Those meeting the criteria reported their symptoms through the digital screener and scheduled a telemedicine consultation. Patients who reported symptoms of a complicated UTI or vaginal infection were able to schedule a telemedicine consultation. Immediately following their consultation, patients received a satisfaction questionnaire. After 7 days, patients received a follow-up questionnaire to assess their symptom resolution. All patients who reported unresolved symptoms received follow-up telemedicine services. Fig 1 shows the participant selection process for this cohort study.

### Physician information and the clinical protocol

The median number of consultations was 79 (IQR = 623) among 84 physicians. Physician specialties varied and included 51.2% (n = 43) family medicine, 29.8% (n = 25) internal medicine, 11.9% (n = 10) emergency medicine, and 4.8% (n = 4) preventative medicine, as well as one physician who was trained in both internal and emergency medicine and one physician who was trained as a radiologist. A clinical protocol provided guidance for physicians to diagnose and treat uncomplicated UTIs using the telemedicine platform. The protocol outlined contraindications, considerations for treatment, and recommended antibiotic regimens (see S1 Table) [18, 22, 26]. All physicians were trained on the protocol, which was available for reference during consultations. The protocol recommended that patients with symptoms of complicated UTIs or vaginal infection be referred to in-person care. However, physicians were permitted to use their discretion to treat via telemedicine or refer to in-person care. STIs were ruled out during physician consultation based on answers provided on the questionnaire. If providers were unsure about treating a patient, they could escalate the issue to the telemedicine program directors for additional guidance.

### Measures

#### Demographic characteristics

Patients were asked their age (in years) and sex during the digital screening process before the UTI consultation. The first three digits of each patient’s zip code were used to determine geographic location.

#### Symptom presentation groups

Patients reported the presence of the following four uncomplicated UTI symptoms: urinary frequency, urinary urgency, dysuria (pain or burning while urinating), and hematuria (blood in the urine). Patients reported the presence of the following complicated UTI symptoms: an oral temperature greater than 99.5 °F (37.5 °C); new onset of nausea and/or vomiting; new onset of feelings of general achiness/feeling unwell; back, abdominal, side, or groin pain; international travel within the past six months; and dehydration within the past two to three weeks [5, 27, 28]. Patients identified the presence of any vaginal infection symptoms: green, yellow, clear, or white discharge; odor from urine/discharge; and vaginal itching, blisters, lesions, or rash. Patients were grouped into three categories according to their reported symptom presentation: uncomplicated UTI symptoms, complicated UTI symptoms, and other (see Table 1). Appointments for recurrent infection (i.e., at least two appointments in six months, aside from relapse, or at least three appointments in 12 months, aside from relapse) were considered a complicated UTI, as long as the patient reported UTI symptoms (even if the patient had reported only symptoms of uncomplicated UTI during their recurrent visits) [19]. The presence or absence of vaginal symptoms was also assessed.

**Table 1:** Symptom groups and descriptions.

Symptom Group	Description	N (%) (N = 51,474)
Uncomplicated UTI^1^^,^^3^	Patients reporting ≥ 2 of the 4 UTI symptoms, with or without symptoms of vaginal infection Not a recurrent infection (first infection in 6 months, or < 3 infections in 12 months)	31,730 (61.6)
Complicated UTI^2^^,^^3^	Patients who reported ≥ 1 of the 4 UTI symptoms AND ≥ 1 symptom of complicated infection, with or without symptoms of vaginal infection (regardless of recurrent infection) OR Any patient with recurrent infection (≥ 2 infections in 6 months or ≥ 3 infections in 12 months) with or without symptoms of vaginal infection (regardless of having only uncomplicated UTI symptoms)	18,788 (36.5)
Other^3^	Patients reporting ≤ 1 of the 4 UTI symptoms, without symptoms of complicated infection, with or without symptoms of vaginal infection Patients reporting none of the 4 UTI symptoms with ≤ 1 of the complicated infection symptoms, with or without symptoms of vaginal infection Regardless of recurrent infection	956 (1.9)

UTI, urinary tract infection.

^1^Uncomplicated UTI symptoms consisted of urinary frequency, urinary urgency, dysuria, and hematuria.

^2^Complicated UTI symptoms consisted of an oral temperature greater than 99.5 °F (37.5 °C); new onset of nausea and/or vomiting; new onset of feelings of general achiness/not feeling well; back, abdominal, side, or groin pain; international travel within the past 6 months; and dehydration within the past 2–3 weeks.

^3^Symptoms of vaginal infections consisted of green, yellow, clear, or white discharge; odor from urine or discharge; and vaginal itching, blisters, lesions, or rash.

#### Prescription use and antibiotic type

Prescription use and antibiotic type were measured by analyzing the number of appointments that resulted in prescriptions and the type and number of medications that were prescribed. A standard clinical protocol was developed based on guidelines for uncomplicated UTIs; thus, the recommended antibiotics were nitrofurantoin, trimethoprim-sulfamethoxazole, and fosfomycin (see S1 Table) [18, 22, 26]. No standardized protocol was developed for complicated UTIs and/or symptoms of vaginal infection. Physicians were permitted to manage these cases according to their evaluation and discretion. However, specific population-guided protocols were developed (e.g., for women older than 65 years, patients with dual infections).

#### Symptom resolution

Patients received a survey seven days after their appointment asking them to indicate whether their symptoms had resolved.

#### Treatment failure and relapse

Appointments that occurred within 30 days after an initial appointment were considered follow-up visits and reported as treatment failure or relapse. These visits were excluded from the original analyses and were instead reported as outcomes of the initial appointment. Due to insufficient data (e.g., it is unclear if most patients experienced any symptom resolution between appointments), treatment failure and relapse were reported together because these groups were indistinguishable.

### Statistical methods

Descriptive statistics were used to summarize the study sample, including frequencies, means, SDs, medians, and IQRs. Specifically, data were presented to examine prescription use and symptom resolution by age and US census region, as well as by grouping those with uncomplicated UTIs, complicated UTIs, and symptoms of vaginal infection. Number of prescriptions received, type of prescription, symptom resolution, and number of treatment failures/relapses by symptom profile category were also assessed. Symptom resolution rates were compared, using chi-square tests, between the complicated and uncomplicated UTI symptom groups and between all women who received a prescription medication with symptom resolution among eight comparator studies, for a total of nine tests [14, 29–35]. Bonferroni correction was used to account for multiple tests, and p-values <0.005 were considered significant. All descriptive statistics and chi-square tests were calculated in R (version 4.0.5) for Macintosh.

## Results

The analysis set (n = 51,474) included data from appointments with patients between the ages of 18 and 84 years (mean = 39.4, SD = 14.7). Most patients were between the ages of 22 to 46 years (n = 30,423, 59.1%), and the South census geographic region had the greatest representation (n = 20,172, 39.2%; see Table 2).

**Table 2:** Patient characteristics^1^.

Characteristics	N (%) (N = 51,474)
Age (years)	
18–21	3,791 (7.4)
22–45	30,423 (59.1)
46–65	14,449 (28.1)
≥66	2,811 (5.5)
Region (US Census Region)^2^	
South	20,172 (39.2)
West	17,851 (34.7)
Midwest	9,970 (19.4)
Northeast	3,481 (6.8)

^1^Raw data counts by age and geographic region for each appointment. Individuals who used the program multiple times appear more than once in this table.

^2^Regions were determined via the US Census regions. West: Alaska, Arizona, California, Colorado, Hawaii, Idaho, Montana, Nevada, New Mexico, Oregon, Utah, Washington, Wyoming. Northeast: Connecticut, Maine, Massachusetts, New Hampshire, New Jersey, New York, Pennsylvania, Rhode Island, Vermont. Midwest: Illinois, Indiana, Iowa, Kansas, Michigan, Minnesota, Missouri, Nebraska, North Dakota, Ohio, South Dakota, Wisconsin. South: Alabama, Arkansas, Delaware, District of Columbia, Florida, Georgia, Kentucky, Louisiana, Maryland, Mississippi, North Carolina, Oklahoma, South Carolina, Tennessee, Texas, Virginia, West Virginia.

### Symptom presentation

Overall, symptom prevalence was high for urinary frequency (n = 48,574, 94.4%), urinary urgency (n = 48,662, 94.5%), and dysuria (n = 50,244, 97.6%), while fewer patients reported hematuria (n = 8,381, 16.3%). Most appointments (n = 31,730, 61.6%) consisted of patients with uncomplicated UTI symptoms, but a substantial number (n = 18,788, 36.5%) reported complicated UTI symptoms (see Table 1). The remaining appointments (n = 956, 1.9%) consisted of patients in the other symptoms group. In total, 9,894 (19.2%) reported at least one symptom of vaginal infection: 5,142 (16.2%) in the uncomplicated UTI symptoms group, 4,618 (24.6%) in the complicated UTI symptoms group, and 134 (14.0%) in the other symptoms group.

### Prescription use and antibiotic choice

Patients from 50,826 (98.7%) appointments received at least one prescription, with 2,818 (5.5%) receiving more than one. Patients from 44,304 (86.1%) appointments received one antibiotic, 2,320 (4.5%) received at least one antibiotic and an additional medication (e.g., phenazopyridine or fluconazole), 498 (1.0%) received two or more antibiotics without an additional medication, and 5 (<0.01%) received one or more other medications without an antibiotic. Prescription data were unavailable for 3,966 (7.7%) patient appointments on the platform.

Overall, adherence to the clinical protocol for recommended prescribing patterns was high (n = 43,932, 94.0%), and patients were most likely to receive prescriptions for antibiotics that were recommended in the clinical protocol (see Table 3). This pattern was consistent across patient groups: uncomplicated UTI symptoms (n = 27,128, 94.4%), complicated UTI symptoms (n = 16,070, 93.7%), and other symptoms group (n = 772, 88.0%). Most women who reported at least one vaginal symptom, in addition to UTI symptoms, received a recommended antibiotic (n = 8,396, 84.9%).

**Table 3:** Symptom groups and prescription counts.

Symptom Group	Received a Prescription^1^ N (%)	Unknown Prescription^2^ N (%)	Received Antibiotic N (%)^3^	Received “Recommended” Antibiotic N (%)^4^	Nitrofurantoin N (%)^5^	TMP-SMX N (%)^5^	Fosfomycin N (%)^5^	Ciprofloxacin (Regular or XR) N (%)^5^^,^ ^6^
Total N = 51,474 (100%)	50,826 (98.7)	3,699 (7.2)	46,754 (92.0)	43,932 (94.0)	24,581 (52.6)	19,404 (41.5)	23 (0.0)	2,431 (5.2)
UTI N = 50,518 (98.1%)	49,903 (98.8)	3,657 (7.2)	45,877 (91.9)	43,160 (94.1)	24,200 (52.7)	19,010 (41.4)	23 (0.0)	2,346 (5.1)
Uncomplicated N = 31,730 (62.8%)	31,431 (99.1)	2,471 (7.8)	28,727 (91.4)	27,128 (94.4)	15,235 (53.0)	11,913 (41.5)	15 (0.0)	1,402 (4.9)
Complicated N = 18,788 (37.2%)	18,472 (98.3)	1,186 (6.3)	17,150 (94.8)	16,070 (93.7)	8,965 (52.3)	7,097 (41.4)	8 (0.0)	944 (5.5)
Other N = 965 (1.9%)	923 (95.6)	42 (4.4)	877 (95.0)	772 (88.0)	381 (43.4)	394 (44.9)	0 (0.0)	85 (9.7)

TMP-SMX, trimethoprim-sulfamethoxazole; UTI, urinary tract infection; XR, extended-release.

^1^Patients who received >1 prescription are only included once in this column.

^2^Some patients received a prescription, but there was no information about the prescription available in the dataset.

^3^Percentage represents the number of patients who received ≥1 antibiotics out of the total number who received a prescription. Patients who received >1 type of antibiotic are only included once.

^4^A recommended antibiotic is defined by the clinical protocol, not by the patient’s self-reported symptoms, and include nitrofurantoin, TMP-SMX, and fosfomycin. The percentage represents the number of patients who received a recommended antibiotic out of the total number who received an antibiotic prescription. Note that 76 people received a prescription for nitrofurantoin and TMP-SMX; they are only listed once in this column.

^5^The percentages represent the number of patients who received that prescription out of the number of patients who received an antibiotic.

^6^Ciprofloxacin was an alternative regimen in the clinical protocol.

### Symptom resolution

**Fig 2:**
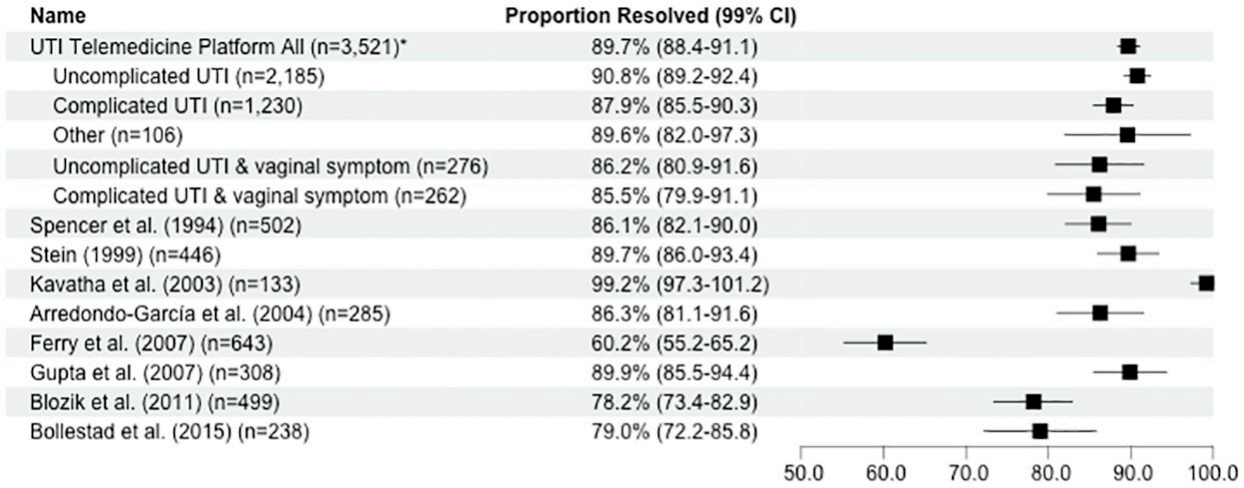
Forest plot of symptom resolution rates. Resolution rates and 99% confidence intervals among all consultations, reported by UTI symptom group from this study and eight previously published comparator studies. “UTI Telemedicine Platform All” refers to all consultations with associated seven day follow-up symptom resolution data, followed by each symptom category. Among the comparator studies, symptom resolution was measured on different days, including: 3 days [35], 4 days [14], 4 to 7 days [31], 5 to 9 days [32, 34], 7 days [30], 8 to 10 days [33], and 9 to 15 days [29]. Of the 8 comparator studies, 7 used in-person standard-of-care [14, 29–34] and 1 used telemedicine services [35]. If the comparator study was a clinical trial with a control group, then symptom resolution rates were only compared with treatment groups who received antibiotics [14]. *“UTI Telemedicine Platform All” refers to the current study.

Symptom resolution data from the seven day follow-up survey were available for 3,541 women, including 2,193 women in the uncomplicated UTI group, 1,241 women in the complicated UTI group, and 107 women in the other symptoms group. Across all groups, 99.4% of patients (n = 3,521) received a prescription. The majority of these patients reported seven day symptom resolution (89.7%, n = 3,160) compared with less than half of patients who did not receive a prescription (40.0%, n = 8; see Table 4). The symptom resolution rate in the complicated UTI group was lower than that of the uncomplicated UTI group; however, this difference did not reach statistical significance (see Fig 2). Among those with a UTI and at least one symptom of vaginal infection, the symptom resolution rates were similar for those with uncomplicated UTIs (86.2%) and complicated UTIs (85.5%). Across all groups, the symptom resolution rate among patients who received a prescription within this study was significantly higher than four of the eight comparator studies (χ^2^ = 20.0–374.1, p<0.001), significantly lower than one study (χ^2^ = 11.9, p<0.001), and not significantly different from the remaining three studies (see Fig 2).

**Table 4:** Symptom resolution stratified by symptom group and overall prescription^1^.

Symptoms	N (%)												
	Uncomplicated UTI Symptoms (n = 2,193)		Complicated UTI Symptoms (n = 1,241)		Other Symptoms (n = 107)		Uncomplicated UTI & Vaginal Symptom (n = 276)		Complicated UTI & Vaginal Symptom (n = 262)		Total		
	Rx	No Rx	Rx	No Rx	Rx	No Rx	Rx	No Rx	Rx	No Rx	Rx	No Rx	Total
Resolved	1,984 (90.8)	4 (50.0)	1081 (87.9)	4 (36.4)	95 (89.6)	0 (0.0)	238 (86.5)	0 (0.0)	223 (86.4)	1 (25.0)	3,160 (89.7)	8 (40.0)	3,168 (89.5)
Unresolved	201 (9.2)	4 (50.0)	149 (12.1)	7 (63.6)	11 (10.4)	1 (100.0)	37 (13.5)	1 (100.0)	35 (13.6)	3 (75.0)	361 (10.3)	12 (60.0)	373 (10.5)
Total	2,185	8	1,230	11	106	1	275	1	258	4	3,521	20	3,541

Rx, prescription; UTI, urinary tract infection.

^1^“Rx” includes individuals who were prescribed any prescription medication.

### Treatment failure and relapse

Overall, 1,156 (2.2%) patients returned to use the program within 30 days after their original consultation: 264 patients (22.8%) returned within seven days, 328 patients (28.4%) within eight to 14 days, and 564 patients (48.8%) between 15 and 30 days after the initial appointment.

## Discussion

To the authors’ knowledge, this is the largest analysis of real-world data from a telemedicine service for treating UTIs to date. Data from 51,474 visits over four years indicated that patients from 98.1% of appointments self-reported at least two symptoms of uncomplicated or complicated UTIs, with dysuria being the most commonly reported symptom. More than half (52.4%) of the sample reported at least one symptom of complicated UTI or vaginal infection. Most appointments (98.7%) resulted in a prescription, and prescription use and type were similar regardless of patient-reported symptoms (uncomplicated UTI, complicated UTI, or vaginal infection). Most patients (94.0%) with available prescription data received antibiotics that were recommended in the protocol (nitrofurantoin, trimethoprim-sulfamethoxazole, and fosfomycin).

Of patients with available follow-up survey data, 89.5% reported symptom resolution, and resolution rates were similar between uncomplicated and complicated UTI groups. Furthermore, the symptom resolution rate across all UTI categories was similar to previous research on UTI telemedicine programs evaluating only uncomplicated UTI patients. Only 2.2% of patients returned to the program within 30 days after an initial appointment, suggesting low treatment failure and relapse rates. Resolution rates among uncomplicated and complicated UTIs were also similar to, or better than, previous reports of in-person standard of care (see Fig 2). Taken together, this analysis demonstrates the effectiveness of large-scale UTI telemedicine programs and extends those observations to include patients who self-report symptoms of complicated UTIs or vaginal infections. Although this study made no direct measurements regarding safety, a retrospective study evaluating the safety and efficacy of telemedicine management of uncomplicated UTIs in 526 women reported that tele-prescription of antibiotics is as safe as prescriptions initiated during in-person consultations [35]. Overall, 78% of patients reported complete symptom resolution three days following teleconsultation, while 14% reported a decrease in uncomplicated UTI symptoms. Four percent reported deterioration, such as increased pain, flank pain, or fever, and 5% reported antibiotic adverse effects [35].

Previous research has reported that women perform well at self-diagnosing UTIs [36]. Despite self-reporting symptoms of complicated UTIs or vaginal infections, these data support the notion that the women using this telemedicine program were accurately able to self-identify that they had a UTI. Furthermore, through discussions with physicians, these patients may have clarified the presence and severity of complicated UTIs or vaginal symptoms, which may have emboldened physicians to provide care for women who self-reported these symptoms at intake. Women who self-reported complicated UTI and vaginal symptoms had similar resolution rates compared with those who reported uncomplicated UTIs symptoms. Thus, these data suggest that a telemedicine approach to identification and treatment of more complicated UTIs and vaginal symptoms may be effective. Further work to determine appropriate sub-grouping and standardized protocols for these conditions is likely warranted. Of note, if the program had excluded patients who reported complicated UTI or symptoms of vaginal infection, 24,064 women (approximately half of the sample) would have required in-person care, either through primary care, urgent care, or emergency department visits.

At scale, telemedicine programs that include uncomplicated and complicated UTIs could be a complementary offering at primary care clinics. This complementary service may result in reduced provider burdens within those settings, as well as within urgent care or emergency departments, freeing up valuable resources, without compromising quality of care or resolution rates. Undeniably, telemedicine access for UTI assessment and treatment can lessen the patient burden of in-person care. Future studies may identify the most effective protocols for integrating such services into the existing primary care setting, or even into urgent care centers and emergency departments.

Another noteworthy outcome of this analysis was that telemedicine physician adherence to recommended antibiotic protocols was higher than previously reported, according to concordance with the Infectious Diseases Society of America guidelines for antibiotics to treat UTIs [37, 38]. The telemedicine physicians in this sample were adherent to these guidelines for 94.0% of appointments that resulted in antibiotic prescriptions. This high adherence rate may have been due to the implementation of the protocol and ease of use [39]. Moreover, provider prescribing practices were regularly audited to assure compliance. The recommended antibiotics in the clinical protocol were based on the guidelines for patients with uncomplicated UTIs [26, 40]. Although not detailed in the current clinical protocol, the recommended antibiotic regimens may differ for patients exhibiting symptoms of pyelonephritis or complicated infection [40]. Telemedicine programs that allow patients with symptoms of complicated UTIs or vaginal infections to receive appointments should include specific instructions for assessing the severity of the infection, the potential need for STI testing, and other medications that should be considered if a UTI diagnosis is not suspected.

### Limitations

As with all real-world evidence, the data used in this analysis were limited by what was collected through the clinical program. The present analysis relied on inferred diagnosis determined from digital responses and patient-reported symptoms at intake. Diagnoses made during treatment consultations were not obtained. As telemedicine programs become more ubiquitous, incorporating post-encounter diagnosis codes would provide greater granularity and understanding of treatment patterns and outcomes, particularly for those with more complicated cases or other symptoms that may be addressed as part of the telemedicine encounter. Integrating these types of programs into electronic health records diagnosis information would allow researchers to identify patients who inaccurately report their symptoms. Future work is needed to determine discrepancies between patient-reported symptoms on intake and physicians’ diagnoses based on direct patient encounters. This knowledge would help to expand clinical protocols to include guidance for determining the risk levels and appropriate next steps for patients who report symptoms of complicated UTI or vaginal infection.

The current analysis evaluated symptom resolution data for a total of 3,541 appointments, representing only 6.9% of the population. Although this still represents a large cohort in both the uncomplicated and complicated UTI groups, we can only speculate a similar rate of symptom resolution over the entire sample population. Notably, patients who did not experience symptom resolution were offered additional care, including referral to in-person care, and as such no symptom resolution data may have been available. A small percentage of patients (2.2%) returned to use the program within 30 days after the initial consultation, suggesting that most patients experienced a full recovery or sought in-person/other care, a feature this analysis cannot resolve. Patient follow-up data are needed to better understand symptom resolution when using direct-to-consumer telemedicine services. Future research may provide insight into how to ensure patients self-report symptom resolution, or perhaps, how data from electronic health records may be used to assess follow-up engagement with in-person care services.

## Conclusions

Telemedicine services make it easier and faster for patients to receive treatment for UTIs by eliminating travel and waiting time at emergency rooms. This analysis demonstrates that telemedicine programs are an effective and scalable option for treatment of UTIs, even for women who self-report symptoms of complicated UTIs or vaginal infection. Most patients in the program exhibited symptoms of uncomplicated or complicated UTIs. Of those patients with available follow-up data, most experienced symptom resolution within seven days after their appointment. Implementing and scaling UTI telemedicine programs and including women who self-report symptoms of complicated UTI or vaginal infection can reduce the burden on urgent care facilities and emergency departments, without compromising quality of care.

## Supporting information

S1 Table Prescription information. (DOCX)
