# Supplementary material for: Real-world evidence: Telemedicine for complicated cases of urinary tract infection
Source: PLoS One. 2023 Feb 2;18(2):e0280386. doi: 10.1371/journal.pone.0280386 (PMC9894494; doi:10.1371/journal.pone.0280386)
Supplement: S1 Table — (DOCX) [file pone.0280386.s001.docx]

Real-world evidence: Telemedicine for complicated cases of urinary tract infection

Short Title: RWE for telemedicine and complicated UTI

Natalie M. Daumeyer^1^, Daniel Kreitzberg^1^, Kathleen M. Gavin^1,2^, Timothy A. Bauer^1,2,*^

^1^Everly Health, Inc., Austin, TX, USA

^2^University of Colorado Anschutz Medical Campus, Aurora, CO, USA

# Supporting information

**S1 Table.** **Prescription information**

| **Recommended Regimens** | | |
| --- | --- | --- |
| Nitrofurantoin  100 mg, twice per day for 5 days | Trimethoprim-sulfamethoxazole 160–800 mg twice per day for 3 days | Fosfomycin  3 g single dose |
| **Alternative Regimens** | | |
| Ciprofloxacin^*^  250 mg twice per day for 3 days | Ciprofloxacin extended release^*^  500 mg once per day for 3 days | Levofloxacin^*^  250 mg once per day for 3 days |
| Ofloxacin^*^  400 mg single dose | Amoxicillin-clavulanate^✝^  500–125 mg twice per day for 3–7 days | Cefpodoxime^✝^  100 mg twice per day for 3–7 days |
| Cefdinir^✝^  300 mg twice per day for 3–7 days | Cefaclor^✝^  250 mg or 500 mg 3 times per day for 3–7 days | Cefadroxil^✝^  500 mg twice per day for 3–7 days |

^*^Fluoroquinolones

^✝^Beta lactams
